# Combining actigraphy and experience sampling to assess physical activity and sleep in patients with psychosis: A feasibility study

**DOI:** 10.3389/fpsyt.2023.1107812

**Published:** 2023-02-23

**Authors:** Lydia E. Pieters, Jeroen Deenik, Sabine de Vet, Philippe Delespaul, Peter N. van Harten

**Affiliations:** ^1^Psychiatric Center GGz Central, Research Department, Amersfoort, Netherlands; ^2^Faculty of Health Medicine and Life Sciences, Department of Mental Health and Neuroscience, Maastricht University, Maastricht, Netherlands; ^3^Mondriaan Mental Health Center, Heerlen, Netherlands

**Keywords:** schizophrenia, motor behavior, accelerometry, ecological momentary assessment, severe mental illness

## Abstract

**Background:**

Sleep disorders and reduced physical activity are common in patients with psychosis and can be related to health-related outcomes such as symptomatology and functioning. Mobile health technologies and wearable sensor methods enable continuous and simultaneous monitoring of physical activity, sleep, and symptoms in one’s day-to-day environment. Only a few studies have applied simultaneous assessment of these parameters. Therefore, we aimed to examine the feasibility of the simultaneous monitoring of physical activity, sleep, and symptoms and functioning in psychosis.

**Methods:**

Thirty three outpatients diagnosed with a schizophrenia or other psychotic disorder used an actigraphy watch and experience sampling method (ESM) smartphone app for 7 consecutive days to monitor physical activity, sleep, symptoms, and functioning. Participants wore the actigraphy watch during day and night and completed multiple short questionnaires (eight daily, one morning, and one evening) on their phone. Hereafter they completed evaluation questionnaires.

**Results:**

Of the 33 patients (25 male), 32 (97.0%) used the ESM and actigraphy during the instructed timeframe. ESM response was good: 64.0% for the daily, 90.6% for morning, and 82.6% for evening questionnaire(s). Participants were positive about the use of actigraphy and ESM.

**Conclusion:**

The combination of wrist-worn actigraphy and smartphone-based ESM is feasible and acceptable in outpatients with psychosis. These novel methods can help both clinical practice and future research to gain more valid insight into physical activity and sleep as biobehavioral markers linked to psychopathological symptoms and functioning in psychosis. This can be used to investigate relationships between these outcomes and thereby improve individualized treatment and prediction.

## 1. Introduction

Disruptions in physical activity and sleep are common in people with schizophrenia spectrum disorders and have been recognized as promising markers that could be related to symptomatology, social functioning, quality of life, and illness course. People with schizophrenia spectrum disorders lack physical activity, which is not only a major contributor to poor physical health and premature mortality (1, 2), but is also strongly related to negative symptoms, cognitive impairment, and quality of life (3–6). Sleep disorders, including insomnia, obstructive sleep apnoea, periodic limb movement disorder, and disruptions in circadian rhythm, are reported in up to 80% of patients with schizophrenia (7). They have been directly related to positive and negative symptomatology, cognitive deficits, and poor psychosocial functioning in patients with schizophrenia spectrum disorders (8–10).

Considering these clinically relevant relationships, improved assessment of physical activity and sleep patterns as biobehavioral markers in psychosis is highly needed (11, 12). Especially, since self-reported and clinician-rated measures are influenced by recall bias and interpretation differences. Recent advancements in technology-based assessments including actigraphy and smartphone-based applications enable promising, easy-to-use, and affordable solutions for monitoring symptoms continuously.

Actigraphy is a non-invasive method, mostly worn as an actigraphy monitor on the wrist, that allows for continuous and precise assessment of sleep patterns (e.g., total sleep duration, sleep onset latency, sleep efficiency, fragmentation, and circadian rhythm) and physical activity (e.g., activity counts, duration of activity, activity intensities, and sedentary behavior) (11). Several studies have proven the validity and reliability of actigraphy for the measurement of sleep and physical activity in healthy individuals and patients with a psychiatric disorder (11, 13–16). For the assessment of physical activity, actigraphy has proven higher validity compared to self-administered retrospective questionnaires, which tend to overestimate total physical activity and underestimate sedentary behavior (15, 17). For the assessment of sleep, wrist actigraphy has shown moderate agreement with polysomnography, although actigraphy tends to overestimate total sleep time and sleep efficiency, especially in patients with a chronic condition (14). Several studies on patients with schizophrenia used actigraphy for the assessment of motor activity (4, 18–22) or sleep (23–25) and linked them to psychotic symptoms or neurobiological substrates, and a few studies used actigraphy to assess both parameters simultaneously for the examination of sleep–wake cycles (26, 27).

Actigraphy measurement of sleep and motor activity can be combined with the smartphone-based Experience Sampling Method (ESM) to allow the collection of ecologically valid daily-life self-report data. ESM is a validated, digital diary method that consists of multiple repeated measurements throughout the day, using short questionnaires that patients can complete using a smartphone application (28, 29). In this way, ESM offers several advantages over clinical observations or self-rated retrospective questionnaires, by (i) minimizing the potential risk recall and “global evaluation” bias, (ii) allowing ecologically valid assessment of experiences in real-world environments, and (iii) enabling to assess the variation of subjective experiences in the flow of daily life, taking person-environment interactions into account (28, 30). Clinical research has demonstrated the feasibility, usability, and value of ESM in patients with severe mental illness (29, 31).

Combining actigraphy with ESM facilitates the examination of the direct relationships between physical activity, sleep, and symptoms in daily life. Only a few studies used a combination of actigraphy and ESM for the assessment of physical activity or sleep in relation to symptomatology in schizophrenia populations. One study found that actigraphy-measured spontaneous motor activity was an objective readout of apathy in patients with schizophrenia (32). Two other studies demonstrated that actigraphy measures of sleep continuity and/or sleep efficiency were related to greater next-day psychotic symptom severity (33, 34). The combination of actigraphy and ESM offers major opportunities for clinical research, as physical activity and sleep patterns hold promise as clinical markers for psychopathological symptom severity and course of the illness in patients with schizophrenia spectrum disorders (3, 6, 8, 10).

Considering these promising applications of ESM and actigraphy for psychiatry research, it is important to establish the clinical acceptability and feasibility of these methods for psychiatric patients before implementing them into research and clinical practice. This study aims to examine the feasibility of the combination of actigraphy and ESM to measure physical activity, sleep, and daily life symptoms in patients with schizophrenia spectrum disorders and seeks to provide guidance for feature actigraphy/ESM studies.

## 2. Materials and methods

### 2.1. Study population

Patients with a schizophrenia spectrum disorder diagnosis were included from June 2020 to October 2022 in the outpatient departments of the mental health care institution GGz Central, the Netherlands. Inclusion criteria were: (i) age 16–65 years; (ii) a diagnosis of schizophrenia-spectrum disorder, i.e., schizophrenia, schizoaffective, schizophreniform, or brief psychotic disorder according to the fifth edition of the Diagnostic and Statistical Manual of Mental Disorders (DSM-5) (35); (iii) good spoken and written command of the Dutch language; (iv) in possession of a smartphone and capable of using one; and (v) able and willing to provide written informed consent. Exclusion criteria were a major neurological disorder (e.g., epilepsy, Parkinson’s disease, or cerebrovascular disease); medical conditions that severely affect physical activity (e.g., fractures, arthrosis); hearing, reading or other disabilities that impede the use of a smartphone application; a DSM-5 diagnosis of moderate, severe, or profound intellectual disability. DSM-5 diagnosis was confirmed by a board-certified psychiatrist. All diagnoses were discussed between researchers and involved clinicians. The Medical Ethical Committee of Maastricht University approved the protocol.

### 2.2. Procedures

**Figure 1:**
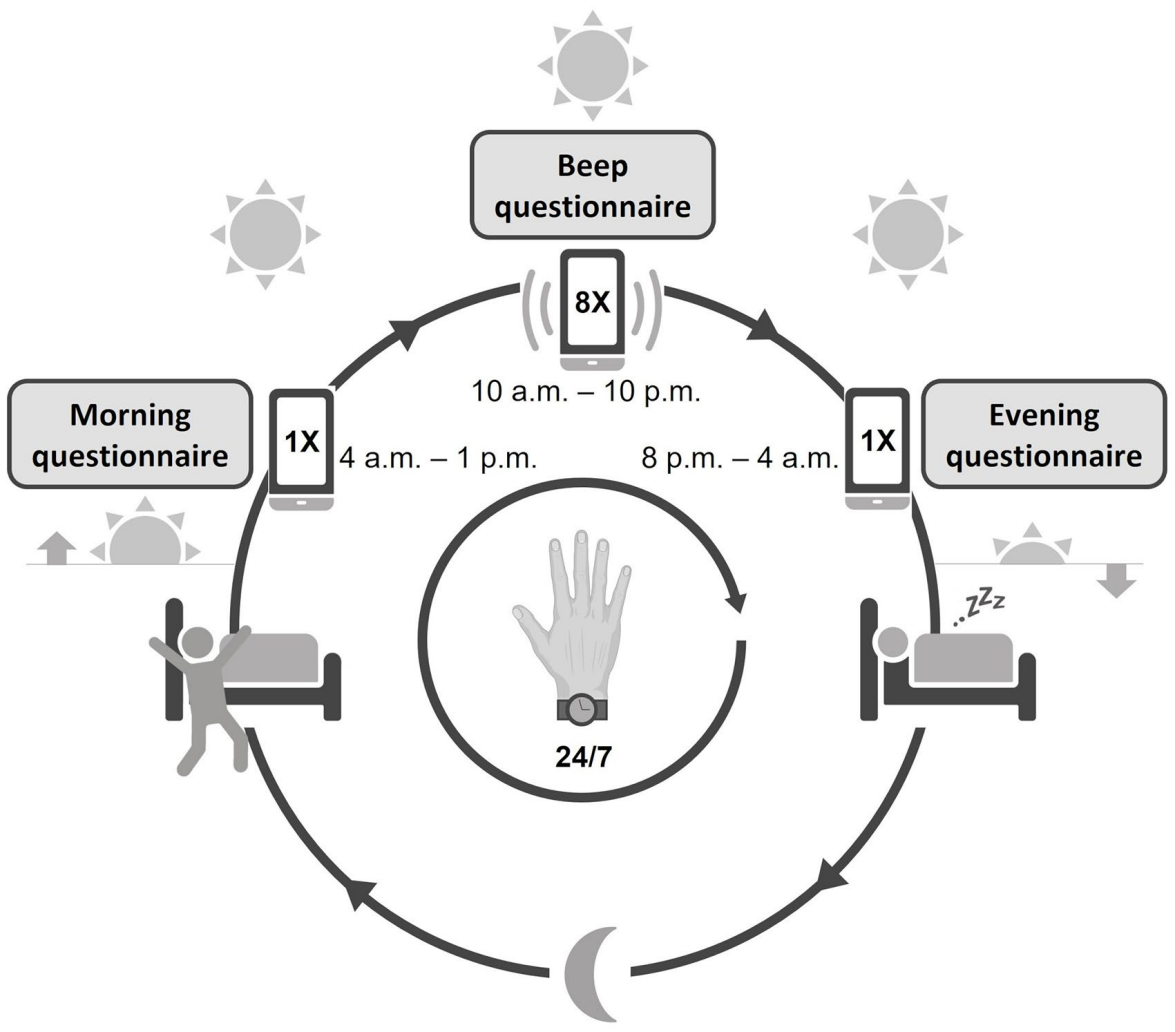
Schematic overview of the study design. The actigraphy watch was worn continuously day and night. The ESM app was used from waking up until going to bed, with a morning, evening, and continuous (beep) questionnaire throughout the day.

A comprehensive assessment of psychopathology, social functioning, quality of life, and neurological functioning was performed, which can be found in the Supplementary material. For the current paper, we chose to present the items that described the main clinical characteristics of the participants and were thus relevant for this feasibility study: demographic and clinical characteristics from medical records, the extended version of the Brief Psychiatric Rating Scale (BPRS) (36), the alcohol and drug use and medical history subscales of the Comprehensive Assessment of Symptoms and History (CASH) (37), the Social and Occupational Functioning Assessment Scale [SOFAS; consisting of the Global Assessment of Functioning (GAF) symptom and handicap subscale] (38), and the self-administered, Manchester Short Assessment of Quality of Life (MANSA) questionnaire (39). All assessments were performed by two researchers (L.P. and S.V.) trained and re-trained with individual instruction sessions and demonstration videos. The baseline visit was followed by a briefing session, in which the participants were instructed to use the ESM app and actigraphy for 7 consecutive days. After completing the ESM and actigraphy data acquisition phase, a debriefing session took place. During this debriefing session, the researcher discussed the ESM and actigraphy results, i.e., physical activity, sleep patterns, and daily life experiences, with the participant. Participants filled out evaluation questionnaires on the ease of use and burden of the actigraphy, the ESM app and the study in general, including items rated on a Likert scale from 1 (“not at all”) to 7 (“very”) and open questions. The evaluation questionnaires for the ESM app and study in general were used in previous feasibility studies of the ESM in patients with schizophrenia (30, 40). The questionnaire that evaluated the use of the actigraphy device was custom-made and designed in the same format as the previous questionnaires. See Figure 1 for a schematic overview of a test day.

### 2.3. Experience sampling method

Participants were instructed to use an ESM smartphone application, Psymate™,^1^ over the same period of 7 days to assess the self-reported, daily fluctuations of mood (positive and negative affect), psychotic experiences, sleep quality, (social) functioning, physical well-being, and their context (activity, location, and company). Participants received a beep-notification on their smartphone at eight semi-randomized time points per day, one beep within every block of 90 min, between 10:00 a.m. and 10:00 p.m. After every beep, participants were asked to fill out questions about current mood, thoughts, context, company, and appraisal of the current situation. A typical question to assess the momentary mood is “I feel cheerful” with 1 = not at all and 7 = very. Participants were instructed to respond to the beep immediately and if they failed to respond within 15 min, the missed questionnaire was skipped. Participants also completed a short morning and evening questionnaire. All questions were short and could be rated quickly, in approximately 2–3 min per assessment, to disturb the flow of normal daily routines only minimally. The ESM questionnaires were based on previous ESM studies in mental health research (41). The ESM questionnaires are enclosed in the Supplementary material.

### 2.4. Actigraphy

Physical activity, sleep, and circadian rhythm were measured by actigraphy using the MotionWatch8 (CamNtech, Ltd., Cambridge, United Kingdom), a wrist-worn device that contains a tri-axial accelerometer that is validated for measuring physical activity and sleep in clinical and non-clinical populations (42, 43). Participants were instructed to wear the device 24 h a day for 7 days and 14 h (the maximum recording length of the device) continuously on the wrist of the non-dominant arm. The length of this observation period was chosen because the current requirements for actigraphy for clinical purposes require recording of at least 72 h and extended monitoring (5 days or longer) reduces the inherent measurement errors in actigraphy and increases reliability (13, 44). Also, capturing both weekdays and weekend days can result in a more complete clinical picture (44). To facilitate sleep and circadian rhythm analysis, participants were instructed to press an event marker before they went to sleep and when they got up. Data were stored at 5-s intervals, to collect almost continuous data needed for the evaluation of movement patterns. Data were analyzed within the proprietary software package (Motionware V1.1.25, CamNtech).

### 2.5. Analysis

In order to assess the feasibility and compliance of the combined assessments of actigraphy and ESM, descriptive analyses of actigraphy and ESM outcomes were conducted. For actigraphy, the wear time within the instructed timeframe was calculated. For ESM, response and completion rates of the ESM morning, evening and daily questionnaires were calculated. To evaluate the burden of each continuous questionnaire, the item “this beep disturbed me” was included in the daily questionnaire. We analyzed means, standard deviations and distributions for this ESM item and the answers on the evaluation questionnaire. All statistical analyses were performed with R version 4.2.0 (45).

## 3. Results

### 3.1. Study population

We included 33 participants, who were mainly male (*n* = 25, 75.8%) and between 25 and 51 years old (*M* = 37.6, SD = 7.8). Patients had relatively low psychiatric symptom severity, with mean BPRS total and positive symptoms scores of 48.4 (SD = 11.7), and 12.9 (SD = 4.5), respectively. The majority of patients were unemployed (*n* = 28, 84.9%) and had serious impairments in social functioning [GAF symptoms mean = 48.4 (SD = 15.0), GAF handicap mean = 47.4 (SD = 12.4)]. Demographic and clinical characteristics are presented in Table 1. Actigraphy and ESM data were obtained in 31participants, as one participant did not use the ESM app and actigraphy watch after completing the baseline visit with the main reason that the ESM measurements would disturb his daily activities (producing music).

**Table 1:** Demographic and clinical characteristics of the participants (*N* = 33).

	
Age (years), mean (SD)	37.6 (7.8)
Sex (male), *n* (%)	25 (75.8)
DSM-5 diagnosis, *n* (%)	
Schizophrenia	16 (48.5)
Schizoaffective disorder	4 (12.1)
Other psychotic disorder^*^	13 (39.4)
Symptom severity	
BPRS total score (range 28–196), mean (SD)	48.4 (11.7)
BPRS positive symptoms (range 7–49), mean (SD)	12.9 (4.5)
BPRS negative symptoms (range 7–49), mean (SD)	12.2 (3.1)
Employment status, *n* (%)	
Employed	5 (15.2)
Unemployed	28 (84.9)
Living situation, *n* (%)	
Alone	16 (48.5)
Sheltered housing	7 (21.2)
With partner/family	10 (30.3)
Functioning	
GAF symptom score (range 0–100), mean (SD)	48.4 (15.0)
GAF handicap score (range 0–100), mean (SD)	47.4 (12.4)

BPRS, brief psychiatric rating scale; DSM-5, diagnostic and statistical manual of mental disorders, fifth edition; GAF, global assessment of functioning; and SD, standard deviation.

* DSM-5 diagnosis of other specified (*n* = 2) or unspecified (*n* = 11) schizophrenia spectrum and other psychotic disorders.

### 3.2. Actigraphy

Participants used the actigraphy watch for a mean duration of 7 days and 1 h (SD = 23 h), within the instructed timeframe of 7 days and 14 h (mean weartime percentage = 93.0%, SD = 12.4, range 47.6–100.0%). One of them did not use actigraphy simultaneously with the ESM app, but, after re-instructions, did wear the actigraphy watch for 3 days following the 7-day period of ESM assessments. Answers from the evaluation questionnaire (ranged 1–7) showed that the wrist actigraphy watch was mostly comfortable to wear during day (mean = 5.1, SD = 1.6, range 1–7) and night (mean = 5.1, SD = 1.5, range 2–7) and did not impair daily activities (mean = 1.9, SD = 1.2, range 1–5). In general, patients were not bothered by wearing the actigraphy watch visible to others (mean = 1.6, SD = 0.7, range 1–3). However, participants were moderately motivated to wear the actigraphy device for a longer period (several months; mean = 3.9, SD = 2.1, range 1–7).

### 3.3. Experience sampling method

The mean completion rates of the ESM were 64.0% (SD = 17.0, range 30.4–91.1) for the continuous questionnaires, 90.6% (SD = 13.9, range 42.9–100.0) for the morning questionnaires and 82.6% (SD = 20.1, range 28.6–100.0) for the evening questionnaires. The ESM item “this beep disturbed me” was rated with a mean of 2.3 (SD = 1.4, range 1.0–5.9) on a scale from 1 to 7. Answers from the evaluation questionnaires showed that participants did not experience difficulties using the ESM application (mean = 1.8, SD = 1.3, range 1–6) or understanding the ESM questions (mean = 2.0, SD = 1.2, range 1–5). Overall, participants found the number (mean = 3.3, SD = 1.8, range 1–7) and duration (mean = 2.5, SD = 1.3, range 1–5) of beep questionnaires not too burdensome.

## 4. Discussion

This study demonstrates that the combination of wrist-worn actigraphy and smartphone-based ESM is a feasible and acceptable method for outpatients with schizophrenia or other psychotic disorder with moderate to serious impairments in social functioning. Participants used the actigraphy watch during the instructed timeframe and completed the majority of the ESM beep questionnaires on their smartphones. Also, they positively evaluated the burden and user-friendliness of ESM and actigraphy.

The application of ESM and actigraphy in populations with psychosis may provide valuable clinical information and new insights for future research and treatment since sleep and physical activity patterns are important biobehavioral features related to psychopathological symptoms and functional outcomes in patients with psychotic and other psychiatric disorders (3–6, 8, 9). Smartphone- and wearable sensor-approaches are rapidly emerging in clinical research. This is the first study that used the combination of actigraphy and ESM to assess physical activity, sleep, and daily life symptoms in patients with schizophrenia spectrum disorders. Several studies have introduced actigraphy for the assessment of sleep or physical activity combined with ESM in patients with schizophrenia, anxiety, and depression (32, 33, 46–48). Furthermore, other methods of wearable sensor data collection have been combined with ESM in schizophrenia and substance-use research, namely continuous electronic physiological monitoring (e.g., electrocardiography) and geolocation (49, 50). Thus, ESM can be complemented by numerous types of data, such as physiological (e.g., cardiac monitoring), environmental (e.g., geolocation, temperature), or behavioral (e.g., actigraphy). In this way, ecologically valid data can be collected on daily-life experiences, biobehavioral symptoms, and environmental factors.

The combined approach of ESM and actigraphy, or other wearable sensors, offers several advantages over ESM alone. Wearable sensors offer the possibility for passive, non-intrusive (not requiring user action) and continuous data collection, contrary to interval-based ESM questionnaires that require the participant’s action and compliance. Although earlier ESM feasibility studies showed that ESM only minimally interfered with daily life routines and had no psychological adverse effects (31, 51, 52), the sampling frequency (i.e., number of questionnaires per day) and duration (i.e., number of days) form the key limitations of ESM research, as higher sampling frequencies and longer sampling duration are practically unrealistic due to the burden on the participant. Complementing ESM with wearable sensor data overcomes these limitations and allows for more intensive or prolonged sampling to answer clinical research questions. Also, the combined ESM and wearable sensor approach allows for the examination of the relations between symptoms and simultaneously measured biobehavioral signs, such as motor activity, sleep, and heart rate (53). Another application for ESM and wearable sensor data is the development of prediction models by machine learning algorithms. These models predict ESM-measured behavior or experiences by a wearable sensor measured biomarker. For example, the prediction of substance-use by GPS-, electrocardiogram-, and actigraphy-data (49), actigraphy and ambient light exposure to predict depressive symptoms (46).

Since mobile and wearable sensor technologies are relatively new in psychiatric research, only a few studies used the combination of ESM and actigraphy in patients with psychotic disorders for either examination of sleep (33, 34) or physical activity (32) in relation to daily symptoms and/or functioning. None of these studies reported user experiences or patient-reported burden. Only one of the studies reported on compliance rates; 77.4% overall response rate of the five beeps per day and the actigraphy device was worn during 97.4% of the instructed timeframe (33). The other studies reported that the overall acceptance was very high and that participants delivered sufficient ESM and actigraphy data.

Previous studies in patients with schizophrenia or other psychotic disorder have validated the feasibility and usability of ESM by itself, without the combination with actigraphy. A study on ESM compliance of 10 pooled ESM studies (*n* = 1,717 individuals with different mental health conditions) that used a paper-and-pencil diary in combination with a beep-producing digital watch (10 beeps per day during 4–6 days) showed that persons with psychosis (i.e., psychotic disorder or psychotic experiences) had response rates of 70%, and were therefore less compliant than healthy participants (83%, *p* < 0.001) (41). An ESM study including 54 outpatients with psychotic disorders using a digital palm computer showed that seven (13%) patients did not fill out the ESM questions at all, and the remaining patients had a compliance rate of 69%, which is comparable to the 64.0% compliance rate in the current study (52). Hospitalized patients with schizophrenia showed higher completion rates 79–81% in ESM studies that also used a palm computer (30, 40). These completion rates are adequate, since completion rates of one-third or higher are traditionally accepted for the collection of ecologically valid ESM data (54). Compared to the aforementioned methods of a digital palm-computer or a paper-and-pencil diary in combination with a digital watch, one would expect higher completion rates and better user experiences with an ESM smartphone app. This was true when researchers started to use smartphones, but now users are overburdened with notification signals and consequently only respond periodically to buffered signals. Often, buffered ESM signals are not active anymore because they exceed the scheduled time to respond. All over the world, smartphone-based ESM response rates are dropping. This may explain the lower completion rates in this study. Other explanations as population, setting, duration and frequency of questionnaires, types of questions, length of questionnaire, and quality of the briefing session may have marginal additional impact on adherence rates (54).

For actigraphy (alone), various studies have demonstrated its usability in populations with psychosis or other severe mental illness, for example, in a large sample (*n* = 184) of long-stay inpatients (18). However, in this relevant study, the aimed measurement of five consecutive days was often not reached and researchers observed challenges in using the devices for a longer period of time.

From a scientific perspective, long-term periods of monitoring with high sampling frequencies seem attractive for the collection of large amounts of data, but burden to the patient, user-experiences and clinical feasibility are often neglected. It is important to establish the optimum ESM and actigraphy intensity and/or duration for the implementation of these methods in psychiatric research and clinical practice. The current feasibility study is in line with ESM guidance papers, where a typical design of 6–7 days with a sampling frequency of 10 questionnaires per day is proposed (29, 31, 54). These specifications were used in previous ESM feasibility and validity studies and were considered sufficient in power analyses that stress that more subjects are more important than more beeps. This study showed that adding actigraphy to ESM under these specifications was feasible and did not put an extra burden on the participants. Thus, the current study provides valuable insight in the feasibility and usability of ESM and actigraphy in patients with psychosis.

### 4.1. Limitations

Some limitations should be noted. First, considering the small sample size of patients with a relatively stable psychiatric condition, the generalizability of the study should be interpreted with caution. However, for the inclusion of patients, no restrictions were made on the stage of the disease, symptom severity or substance abuse, to obtain a diverse sample of patients with schizophrenia spectrum disorders. Furthermore, the low psychotic symptom severity scores in the sample do not necessarily mean that the sample is not representative; understandably, patients who are relatively stable and treatment compliant want to participate in such a study. Therefore, the sample represents patients for whom these novel developments can be used in clinical practice. Furthermore, the stable condition of patients offers the possibility of a longer duration of sampling and intensive measurement of symptomatic, behavioral, and environmental factors. Second, we assessed the feasibility and acceptability of a specific sampling duration (i.e., 7 days) and frequency (i.e., a total of 10 daily questionnaires and actigraphy during day and night), and therefore cannot extrapolate our results to longer durations of monitoring or more intensive sampling frequencies. Previous ESM studies have demonstrated that a sampling duration of up to 10 days with a sampling frequency of 8–10 questionnaires per day is acceptable for the collection of ecologically valid data in psychosis research (28, 29, 31).

### 4.2. Future challenges and directions

The application and interpretation of this “real-world” data for clinical research faces several challenges (53). First, while wearable sensor data claim to be objective and more precise than the regular, clinician-rated, assessment methods, the content validity of wearable sensor data often remains unclear. For example, when actigraphy is compared to polysomnographic measures of sleep, actigraphy tends to overestimate total sleep time and sleep efficiency, especially in patients with a chronic condition (14). However, actigraphy has the major advantage over polysomnography that it can be applied in a patient’s home environment for multiple days, compared to the usually one-day clinical setting of polysomnography measurements. Second, ESM and wearable sensor studies primarily aim observational data collection, although repeated measures with a substantial number of data points can be used to gain insight into causality (e.g., interrupted time series design). The combination of ESM and wearable sensor data collection allows for detecting small or fast changes over time and thereby helps disentangling complex symptom-behavior-environment interactions. Third, although ESM and wearable approaches are non-intrusive and user-friendly, they may not be suitable for all types of populations. For example, one may presuppose that patients experiencing an acute episode of psychosis, elderly patients unfamiliar with mobile devices, or patients with intellectual disabilities can experience difficulties in using these methods. However, ESM studies have shown that these methods are feasible and provide valid and reliable data for the aforementioned populations (55–57). ESM and wearable sensor methods can be adjusted to the patient’s needs and capabilities, e.g., with respect to the question types, intensity and duration of the measurements, and support in using the mobile or wearable devices, and therefore can be used in a wide range of clinical populations.

## 5. Conclusion

The combination of ESM and actigraphy is a feasible and acceptable method for patients with psychosis. It can be used for the collection of real-world, ecologically valid, (semi) continuous data on sleep patterns, physical activity, and daily life symptoms, can offer new and valuable insights into the relationships between sleep, activity, psychopathology, and functioning. As mobile technologies are ubiquitous, also in psychiatric populations, these novel methods can be easily implemented in clinical and research practice and pave the way for psychosis prediction, new (early) intervention targets, and personalized treatment.

## Data availability statement

The raw data supporting the conclusions of this article will be made available by the authors, without undue reservation.

## Ethics statement

The studies involving human participants were reviewed and approved by Medical Ethical Committee of Maastricht University. The patients/participants provided their written informed consent to participate in this study.

## Author contributions

LP, JD, PD, and PH: conceptualization and methodology. LP and SV: data curation, formal analysis, investigation, and visualization. LP, JD, and PH: project administration. PD and PH: resources. JD, PD, and PH: supervision. LP: writing—original draft. JD, PD, PH, and SV: writing—review and editing. All authors contributed to the article and approved the submitted version.

## Conflict of interest

The authors declare that the research was conducted in the absence of any commercial or financial relationships that could be construed as a potential conflict of interest.

## Publisher’s note

All claims expressed in this article are solely those of the authors and do not necessarily represent those of their affiliated organizations, or those of the publisher, the editors and the reviewers. Any product that may be evaluated in this article, or claim that may be made by its manufacturer, is not guaranteed or endorsed by the publisher.

## Supplementary material

The Supplementary material for this article can be found online at: https://www.frontiersin.org/articles/10.3389/fpsyt.2023.1107812/full#supplementary-material

## Footnotes

^1^ www.psymate.eu
