# Supplementary material for: Combining actigraphy and experience sampling to assess physical activity and sleep in patients with psychosis: A feasibility study
Source: Front Psychiatry. 2023 Feb 23;14:1107812. doi: 10.3389/fpsyt.2023.1107812 (PMC9996223; doi:10.3389/fpsyt.2023.1107812)
Supplement: Supplementary file 1 [file Data_Sheet_1.doc]

Supplementary Material

**Combining actigraphy and experience sampling to assess physical activity and sleep in patients with psychosis: a feasibility study**

Lydia E. Pieters^1,2*^, Jeroen Deenik^1,2^, Sabine de Vet^1^, Philippe Delespaul^2,3^, Peter N. van Harten^1,2^

**Supplement 1.** Overview of assessments

**Supplement 2.** Experience Sampling questionnaire

**Supplement 1.** Overview of assessments of the Novel Assessments for Monitoring PSYchosis in DAILY life (PSYDAILY) study.


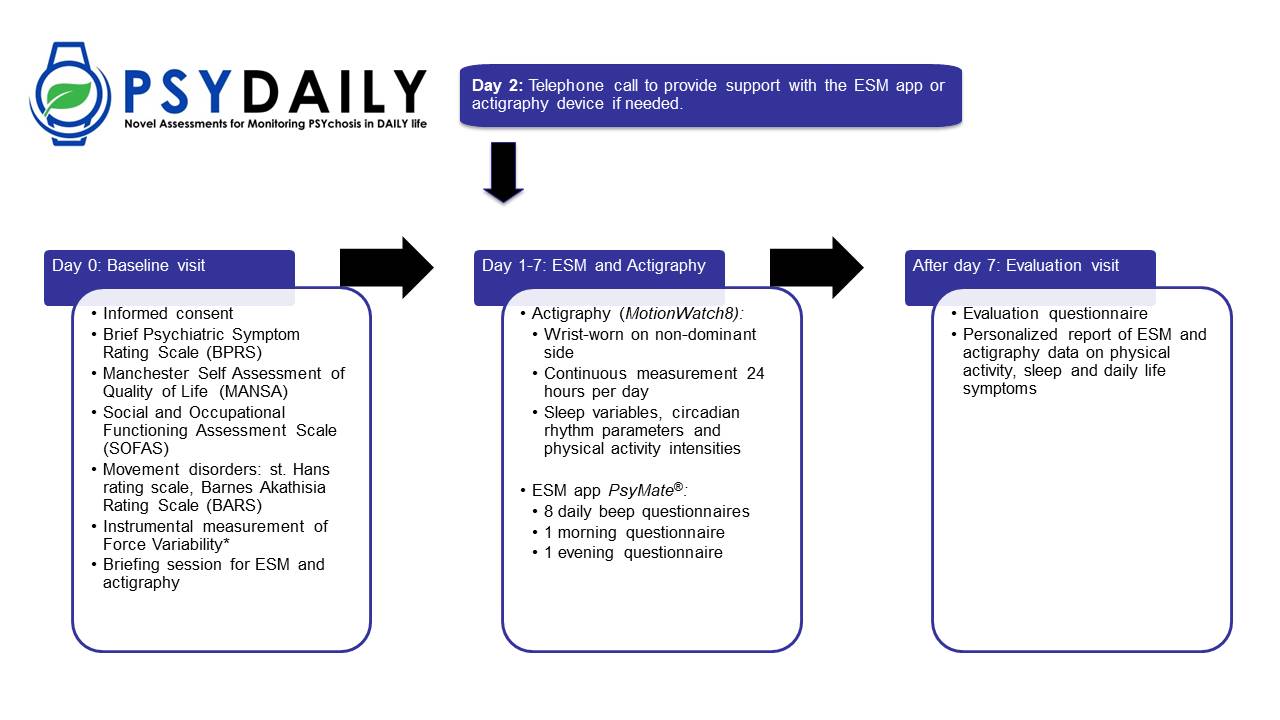


*Instrumental measurement of force variability can be used as a proxy for dyskinesia. See:

Caligiuri, M. P., & Lohr, J. B. (1990). Fine force instability: a quantitative measure of neuroleptic-induced dyskinesia in the hand. *The Journal of Neuropsychiatry and Clinical Neurosciences*, *2*(4), 395–398.

Koning, J. P., Kahn, R. S., Tenback, D. E., van Schelven, L. J., & van Harten, P. N. (2011). Movement disorders in nonpsychotic siblings of patients with nonaffective psychosis. *Psychiatry Research*, *188*(1), 133–137.

**Supplement 2.** Experience Sampling questionnaire, English translation from the original Dutch version. Items were rated on a Likert scale from 1 (not at all) to 7 (very) unless otherwise specified. The beep questionnaire was presented at eight semi-random time points during the day. The morning and evening questionnaires were rated just after or before going to sleep.

**Beep questionnaire**

I feel cheerful

I feel insecure

I feel relaxed

I feel anxious

I feel satisfied

I feel irritated

I feel down

I feel enthusiastic

*Psychopathology:*

I feel suspicious

I feel unreal

I hear voices

I see things

I feel in control

My thoughts are difficult to let go

My thoughts are influenced by others

*Context*

What am I doing? *[work/study, on the road/travelling, sports, having a conversation, housekeeping, self-care, eating/drinking, leisure, resting, other, nothing]*

I would prefer to do something else.

Where am I? *[at work/school, on the road, in a public place, at home, at mental health care institution, somebody else’s home, somewhere else]*

With who am I? *[partner, family, healthcare workers, pets, friends, colleagues, acquaintances, housemates, strangers or others, nobody]*

If alone: I would prefer to be with others. If with others: I would prefer to be alone

*Physical well-being*

I am hungry

I am tired

I am in pain

*Other:*

This beep disturbed me

**Morning questionnaire**

How long did it take before I fell asleep last night? *[0-5 min , 5-15 min, 15-30 min, 30-45 min, 45 min – 1 hr, 1-2 hrs, 2-4 hrs, >4 hrs]*

How often did I wake up last night? *[0 times, 1 time, 2 times, 3 times, 4 times, 5 times, >5 times]*

How long did I lay awake this morning before I got up*? [0-5 min, 5-15 min, 15-30 min, 30-45 min, 45 min – 1 hr, 1-2 hrs, 2-4 hrs , >4 hrs]*

I slept well

I am looking forward to today

**Evening questionnaire**

Today, it was difficult to motivate myself

Today, it was difficult to remember things

Today, it was difficult to get things done

Today, I was able to concentrate

Today, I have been able to look after myself

The items were guided partly by literature on mood assessment in daily life^1^, the Positive and Negative Affect Schedule^2^, and the Consensus Sleep Diary^3^, and partly by previous ESM studies assessing mood, psychotic experiences, sleep and functioning in patients with a psychotic disorder^4–7^.

**References:**

1. Wilhelm P, Schoebi D. Assessing Mood in Daily Life. *Eur J Psychol Assess*. 2007;23(4):258-267. doi:10.1027/1015-5759.23.4.258

2. Watson D, Clark LA, Tellegen A. Development and validation of brief measures of positive and negative affect: the PANAS scales. *J Pers Soc Psychol*. 1988;54(6):1063-1070. doi:10.1037//0022-3514.54.6.1063

3. Carney CE, Buysse DJ, Ancoli-Israel S, et al. The Consensus Sleep Diary: Standardizing Prospective Sleep Self-Monitoring. *Sleep*. 2012;35(2):287-302. doi:10.5665/sleep.1642

4. Mulligan LD, Haddock G, Emsley R, Neil ST, Kyle SD. High resolution examination of the role of sleep disturbance in predicting functioning and psychotic symptoms in schizophrenia: A novel experience sampling study. *J Abnorm Psychol*. 2016;125(6):788-797. doi:10.1037/abn0000180

5. Palmier-Claus JE, Dunn G, Lewis SW. Emotional and symptomatic reactivity to stress in individuals at ultra-high risk of developing psychosis. *Psychol Med*. 2012;42(5):1003-1012. doi:10.1017/S0033291711001929

6. Reininghaus U, Kempton MJ, Valmaggia L, et al. Stress sensitivity, aberrant salience, and threat anticipation in early psychosis: An experience sampling study. *Schizophr Bull*. 2016;42(3):712-722. doi:10.1093/schbul/sbv190

7. Klippel A, Myin-Germeys I, Chavez-Baldini UY, et al. Modeling the Interplay Between Psychological Processes and Adverse, Stressful Contexts and Experiences in Pathways to Psychosis: An Experience Sampling Study. *Schizophr Bull*. 2017;43(2):302-315. doi:10.1093/schbul/sbw185
